# Love Acoustic Wave-Based Devices and Molecularly-Imprinted Polymers as Versatile Sensors for Electronic Nose or Tongue for Cancer Monitoring

**DOI:** 10.3390/s16060915

**Published:** 2016-06-20

**Authors:** Corinne Dejous, Hamida Hallil, Vincent Raimbault, Jean-Luc Lachaud, Bernard Plano, Raphaël Delépée, Patrick Favetta, Luigi Agrofoglio, Dominique Rebière

**Affiliations:** 1 IMS, University Bordeaux, CNRS UMR 5218, Bordeaux INP, 33405 Talence, France; hamida.hallil-abbas@u-bordeaux.fr (H.H.); vraimbau@laas.fr (V.R.); jean-luc.lachaud@ims-bordeaux.fr (J.-L.L.); bernard.plano@ims-bordeaux.fr (B.P.); dominique.rebiere@ims-bordeaux.fr (D.R.); 2 LAAS, CNRS UPR 8001, 31031 Toulouse, France; 3 Normandie Univ., UNICAEN, UNIROUEN, ABTE, 14000 Caen, France; raphael.delepee@unicaen.fr; 4 Comprehensive Cancer Center François Baclesse, UNICANCER, 14076 Caen, France; 5 ICOA, University Orléans, CNRS, CNRS UMR 7311, F-45067 Orléans, France; patrick.favetta@univ-orleans.fr (P.F.); luigi.agrofoglio@univ-orleans.fr (L.A.)

**Keywords:** chemical sensor, surface acoustic wave, MIP, nucleoside, cancer biomarker, urinary, microfluidic, electronic tongue

## Abstract

Cancer is a leading cause of death worldwide and actual analytical techniques are restrictive in detecting it. Thus, there is still a challenge, as well as a need, for the development of quantitative non-invasive tools for the diagnosis of cancers and the follow-up care of patients. We introduce first the overall interest of electronic nose or tongue for such application of microsensors arrays with data processing in complex media, either gas (e.g., Volatile Organic Compounds or VOCs as biomarkers in breath) or liquid (e.g., modified nucleosides as urinary biomarkers). Then this is illustrated with a versatile acoustic wave transducer, functionalized with molecularly-imprinted polymers (MIP) synthesized for adenosine-5′-monophosphate (AMP) as a model for nucleosides. The device including the thin film coating is described, then static measurements with scanning electron microscopy (SEM) and electrical characterization after each step of the sensitive MIP process (deposit, removal of AMP template, capture of AMP target) demonstrate the thin film functionality. Dynamic measurements with a microfluidic setup and four targets are presented afterwards. They show a sensitivity of 5 Hz·ppm^−1^ of the non-optimized microsensor for AMP detection, with a specificity of three times compared to PMPA, and almost nil sensitivity to 3′AMP and CMP, in accordance with previously published results on bulk MIP.

## 1. Introduction

During the recent past, health and life expectancy of people all over the world have become major public concerns, involving obvious links with both diseases detection and treatment, as well as environmental quality as a causal link, among others [1,2]. For instance, air pollution creates a substantial burden of disease, including cardiovascular, oncologic, neurodegenerative, as well as chronic respiratory diseases, reducing life expectancy by almost one year on average [3,4]. Cancers figure among the leading causes of morbidity and mortality worldwide, as published by the International Agency for Research on Cancer (IARC), the specialized cancer agency of the World Health Organization (WHO) [5,6]. During the year 2012, the number of new cases was approximately 14 million, with 8.2 million cancer-related deaths, and the numbers were expected to rise by about 70% over the next two decades. Among men, lung cancer was the first cause, and the prostate was one of the five most common sites, and the second one among men.

These tragic numbers might fall, however, if patients were detected earlier and treated appropriately. Conventional methods used for clinical diagnoses commonly include invasive and potentially hazardous biopsy procedures, endoscopy, mammography, blood tests, microbial culture tests, computed tomography (CT scan), magnetic resonance imaging (MRI), positron emission tomography (PET scan), ultrasonography or X-ray imaging of organs, and DNA homology tests [7]. As more or less highly heavy and invasive techniques, many of these methods not only present some risks of serious adverse side effects, such as surgical complications or internal burns, but they often discourage patients from participating in preventive disease-screening procedures. Furthermore, conventional analytical techniques, such as SPE-HPLC-UV, CE-UV, LC-MS/MS, GC-MS/MS, though offering convenient ways of analyzing complex real samples, they also present some drawbacks and limitations. Additionally, these methods are not amenable to a rapid and routine clinical assay owing to the polar nature of nucleosides and nucleotides and inevitable complex sample pretreatment (extraction, deproteinization, derivatization, *etc*.) before analysis. Furthermore, they need blood collection and sample handling, with possible contamination, and they may require highly-skilled personnel for handling [7]. Finally, they are also time consuming and require sophisticated and expensive instrumentation.

Thus, there is still a crying need for the development of efficient tools in order to pre-empt this alarming problem that threatens public health. In this context, microsensors are expected to offer complementary analytical tools compared to conventional methods, as devices dedicated to specific targets, with short response time and convenience of usage. Among sensing systems under development, some of them aim at improving the environmental quality or at enabling reliable detection of diseases in their early stages, preferably by using non-invasive tools. Both gas and liquids can be useful as sample media, depending on the application targeted.

We present in this paper a short background review on microsensing systems targeting point-of-care (POC) clinical disease diagnoses, both electronic nose (e-nose) and tongue, for detection in gas and liquid media. Then we focus on an innovative approach aiming the non-invasive follow-up care of urinary modified nucleosides as biomarkers from colorectal cancer, by the development of a diagnosis tool based on an acoustic wave biosensor and molecularly-imprinted polymers (MIP) as recognition material [8]. Compared with static measurements reported in [9], the choice of particularly highly-sensitive acoustic modes and the combination with a microfluidic chip aims the real-time detection of tumor markers at low concentrations. The choice of transducing technology based on an acoustic Love Wave (LW) device combined with a recognition film of MIP has been presented previously and will be briefly restated in this paper [10]. More details on specifically-focused materials and methods will be presented, as well as new results related to polymeric films deposition, among them the influence of the film thickness. A particular model of nucleosides was chosen as the main target, as well as three potential interfering molecules as targets, in order to constitute a complete coherent set of results for this study. We describe the detection results in static, then in dynamic modes with the four compounds, are described and analyzed. These results are finally discussed in the perspective of future use, as diagnosis tool and more than that, as a tool for therapeutic drug monitoring (TDM).

## 2. Background Review on Electronic Nose and Tongue for Fast Disease Diagnoses Issues

### 2.1. Gas Media

Recent advancements deal with the use of electronic nose (e-nose) systems, targeting point-of-care clinical disease diagnoses. They are based on the analysis of human odors, with specific pattern betraying the presence of specific volatile biomarkers of specific human diseases, metabolic disorders, and the overall health status of people [7,11,12,13,14,15,16,17]. In this way, it is known that abnormal metabolisms of tumor cells alter the chemical composition of volatile organic compounds (VOCs) released from air expired or in the headspace of body fluids and tissues. VOCs, such as acetone (propanone) [18,19,20,21,22], ammonia (NH_3_) [23,24,25], nitrogen oxides (NO_x_) [26,27,28,29,30], or hydrogen sulfide (H_2_S) [31,32,33,34,35] are among those investigated. Sensors based on metal-oxide semiconductors, typically thin or thick films of tin oxide SnO_2_ (or doped SnO_2_) whose electronic properties are altered by reaction with a target gas [36], are the most commonly used for monitoring gases. Indeed, they offer many advantages, among them compatibility with standard microelectronics fabrication and then low cost [37]. More generally, many sensing technologies are investigated for VOC detection or monitoring, based on electrochemical methods (amperometric, potentiometric, based on field effect transistors (FET) or on chemiresistive material deposited on interdigitated electrodes (IDEs)), direct or indirect optical methods (including infrared (IR), Fourier transform infrared (FTIR), non-dispersive infrared (NDIR), diode laser sensing and ultraviolet (UV) absorption, surface plasmon resonance (SPR)), or using mechanical effect, such as microcantilever or piezoelectric transducer (quartz crystal microbalance (QCM), surface acoustic wave (SAW)). Most of them are presented in [38] which reviews sensor-based methods for monitoring hydrogen sulfide, one of most common organic VOCs, which is carcinogenic, as well as for sensing formaldehyde. A major concern in the practical use of any sensor is the lack of high sensitivity (few ppb) and the selectivity, even more in real samples with complex matrices. Identified methods of improvement are based on the use of (a) multi-functionalization or dopants; (b) an array of sensors, which can be ever enriched by using several transducer technologies; (c) appropriate pattern recognition tools, analyzing both steady-state signals, as well as their transient part when available. The implementation of these combined methods leads to the e-nose approach which is considered as the best route to compensate for the lack of selectivity of individual sensors and also to provide good coverage for multiple types of vapors [39]. E-nose devices are particularly suited for such a field as biomedical application, as they are sensitive to a wide range of VOCs and can effectively distinguish between different complex gaseous mixtures by combining gas sensor arrays and pattern analysis techniques for the detection, identification, or quantification of VOCs [13]. Generally, e-nose contains a set of chemical sensors responding to various gases with different sensitivity and selectivity. The latest scientific studies show that researchers have developed many techniques to select useful sensors from the original sensor array. Simplifying the manufacturing process of the transducer or the choice of a functionalized sensitive layer to increase sensitivity and selectivity may not only improve the performance of the classifier, but also reduce the cost of devices. Several top companies invest in these new markets for sensors and the research and development of new tools dedicated to odor, and especially breath analysis, such as Toshiba [40] or Siemens [41], as such sensor systems could independently detect anomalies in people′s breath that could indicate an illness. In this way, after having detected asthma successfully by measuring the nitrogen monoxide (NO) concentration in breath, Siemens is now developing a system for the early-stage detection of lung cancer, by first finding out a set of molecules that the sensor has to react to so that it can make a definite diagnosis.

### 2.2. Liquid Media

Depending on the type of disease, or of cancer specifically, direct detection in liquid sample (biological fluids or intracellular media) tests can be more relevant. In such way, modified nucleosides, derived mostly from transfer ribonucleic acid (tRNA), have been shown to be excreted in abnormal amounts in the urine of cancer patients [8], which can also be used as body fluid for non-invasive tests. About 20 of them have been already used as tumor markers, for example for breast cancer [42,43,44,45,46,47,48], colon and liver cancer [49,50], or lung cancer [51]. Among these modified nucleosides, are pseudouridine, 1-methyladenosine, 1-methylguanosine, 2-methylguanosine, 1-methylinosine, or 2-pyridone-5-carboxamide-N1-ribofuranoside. They can neither be reutilized nor further degraded, and they are excreted without any salvage pathway, as intact molecules in the urine [52]. Although the urinary excretion of these modified nucleosides is quite low, it betrays the higher turnover rate of tRNA in tumor tissues [53,54,55,56]. Recently, Zhang *et al*. [57] have reported that the intracellular fluctuations of six endogenous nucleotides levels (AMP, UDP, CTP, ATP, UTP, and GMP) were significantly higher in cancer cells. Moreover, the adenine nucleotides, *i.e.*, AMP, ADP, ATP, and adenosine, are signaling molecules related to the modulation of immune responses in cancers [58]. Thus, intracellular nucleotides are considered as potential biomarkers in tumor cells, especially for ATP and UTP.

Different analytical techniques have been reported for the determination of urinary or intracellular modified nucleoside and endogenous nucleotide levels, but their detection is still challenging due to the low level of modified nucleosides as well as to the complexity of the biological matrix such as urine (and to a lesser extent intend, blood) and limitations of conventional analytical techniques. Thus, in a manner similar to electronic nose, several teams worldwide have been working on electronic tongue, combining a setup of several microsensors with data analysis [59,60,61].

## 3. Materials and Methods

### 3.1. Microsensor

#### 3.1.1. Love Wave Transducer

Among microsensing technologies, those based on mechanical effects, such as acoustic wave devices, offer advantages in terms of versatility, especially surface acoustic waves (SAW), which provide high sensitivity to surface effects and reliable component with no moving part. Of all acoustic wave devices, guided shear horizontal surface acoustic wave (SH-SAW) or Love wave (LW) devices appear most promising for biochemical detection in liquid environments: (1) SH-SAW are more sensitive than bulk waves to perturbations produced from the environment without the excessive loss associated with Rayleigh SAW in liquids; (2) the selected piezoelectric materials and transducer designs lead to high Q (quality factor) structures; (3) device frequencies can be scaled to high frequencies of hundreds of megahertz, enabling high sensitivity under conditions of noise reduction; and (4) devices are small, robust, and easy to incorporate into online low-cost systems [62].

The Love wave delay-line was described in previous works [10,63] and its overall shape can be seen as part of the test setup described in Section 3.2. It consists of a dual delay line based on a quartz as piezoelectric substrate, split interdigitated electrodes (40 μm periodicity or wavelength λ) to generate and receive, piezoelectrically, both LW paths, and a 4 μm SiO_2_ guiding layer. These characteristics lead to a 118 MHz synchronous frequency. This dual delay line setup allows differential measurements with a reference, improving the robustness of the platform when the environmental conditions vary during measurements [64].

#### 3.1.2. Molecularly-Imprinted Polymeric Thin Film

In order to ensure specific and even selective interaction for target biomarkers, proteins, such as enzymes, antigens, and antibodies, or DNA, are typically used as recognition biomolecules grafted on the sensor surface, using various attachment strategies. However, these natural receptors are expensive or difficult to produce, with limited lifetime and applicability. Molecular imprinting was developed to overcome these limitations, by creating tailor-made bioreceptors to recognize and rebind a target molecule (substrate) with both high specificity and affinity [65]. Basically, selective imprints are generated by polymerizing a prepolymer in the presence of a template, which is the target molecule or a model one. While the polymer is cured, functional groups in the prepolymer orient toward their counteracting partners in the template and the polymer is cross-linked, resulting in “freezing” the orientation of the functional groups. After extraction of the template, the remaining cavities reproduce the size, shape, and surface chemistry of the template molecules, acting as a molecular memory printed in a three-dimensional polymer network for further rebinding reactions [66,67].

In recent years, due to their high recognition ability, molecular imprinting emerged as a powerful technique for the preparation of various MIP-based sensors due to their long-term stability, high selectivity, and easy preparation. In that case, the MIP membranes must be synthetized in situ at the surface of selected sensors. Several MIP-based sensors were developed to detect either anticancer or antiviral nucleosides/nucleotides in human biological fluids. For instance, Ersoz *et al.* [68] have developed a MIP-QCM sensor in order to determine the level of the 8-OHdG (one of the most abundant oxidative DNA lesions resulting from reactive oxygen species (ROS), in blood serum from a breast cancer patient. The 8-OHdG imprint was based on a methacryloylbasedmetal-chelate polymer (MAAP-Fe(III)). The 8-OHdG level was found as 0.297 μM for MAAP-Fe based QCM sensor, showing the best detection limit than the other 8-OHdG detection methods mentioned in the literature. The anticancer-approved drug gemcitabine was detected in serum samples with a limit of detection of 3 fM by a highly sensitive molecularly-imprinted film formed in situ on gold electrodes via electropolymerization of PATP-functionalized gold nanoparticles [69]. Cyclic AMP (cAMP; adenosine 3′,5′-cyclic monophosphate), a second messenger, is an important intracellular regulator involved in a cascade of events that transduce the signal into changes in many cells. A biomimetic sensor for cAMP was fabricated in combination with an ion-sensitive field-effect transistor (ISFET) as a transducer and a cAMP-imprinted polymer as a molecular recognition material [70,71]. The cAMP-imprinted polymer showed high binding ability to and selectivity for cAMP in aqueous media. The antiviral ganciclovir was quantitate in human serum plasma by a selective and sensitive voltammetric sensor based on electropolymerization of molecularly imprinted polymer with gold nanoparticles (AuNPs) onto multiwalled carbon nanotubes (MWCNTs)/glassy carbon electrode [72]. Similar approach was reported by El Gohary *et al.* for the cyclic voltammetric determination of famciclovir in pharmaceutical preparations where the famciclovir-MIP was synthesized and applied as additive within a carbon paste electrode [73]. Additional electrochemical and QCM sensors, fluoresccent MIP-based chemosensors, were also developed [74].

Part of our research program aimed to determine some colorectal cancer biomarkers, [8], we have turned our attention to the development of a MIP-based SAW-sensor for the detection of various urinary modified nucleosides as well as for the detection of the AMP (adenosine-5′-monophosphate).

**Figure 1:**
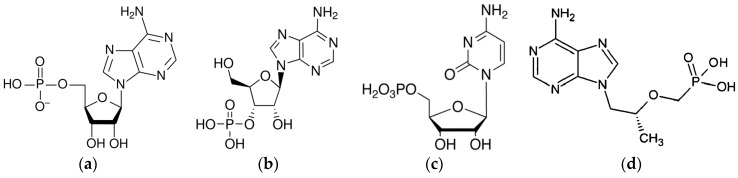
Commercial nucleotides used for rebinding: (**a**) Adenosine-5′-monophosphate (AMP); (**b**) Adenosine-3′-monophosphate (3′-AMP); (**c**) Cytidine-5′-monophosphate (5′-CMP); (**d**) 2-phosphono methoxypropyl adenine (PMPA). Solutions were prepared from buffer solution made of acetic acid/hydroxylamine (AcOH/NH_2_OH 1 mM pH 7) spiked with nucleotide at a concentration 5 mg/L.

The MIP was prepared for a commercial nucleotide adenosine-5′-monophosphate (AMP) as the target model, represented in Figure 1a, with preliminary bulk process optimization to give the best specific binding of AMP towards other nucleotides or deoxy analogues [75].

The step-wise deposition process of thin layers of AMP-based molecularly-imprinted polymer is as follows:

- Piranha cleaning: The LW substrates were cleaned by piranha solution (1:1 (*v*/*v*) concentrated sulfuric acid/30% hydrogen peroxide) to suppress organic and metallic impurities and form an oxide layer at the sensor surface (caution: piranha solution is extremely corrosive and can react severely with organic compounds, gloves, *etc*., which makes it essential that personal protective equipment be used during this step). The substrates were then rinsed thoroughly with 18 Mohm·cm deionized water and dried under a stream of nitrogen [76].
- Surface activation: The sensors were rinsed with toluene and purged overnight in a silane/toluene (2%:1 (*v*/*v*)) mixture and then placed into a laboratory oven at 200 °C for 30 min. The silane promotes covalent attachment of the MIP layer to sensor surface [77].
- Preparing of MIP solution: The AMP-MIP solution preparation process was adapted and optimized for surface coating. It consists of an ionic-noncovalent dual approach in which the best specific binding of AMP was obtained with one equivalent of 2-(dimethylamino)ethyl methacrylate for interaction with the negatively charged phosphate moiety, and ten equivalents of acrylamide to favor interactions with the nucleobase and the sugar moiety [75].
- Thus, 50 mg template AMP was added to 102.3 mg functional monomer acrylamide (AA) and stirred together with 24.2 µL 2-(dimethylamino) ethyl methacrylate (DMAEM) and 1.49 mL ethylene glycol dimethylacrylate (EGDMA), for 5 min. The mixture was dissolved in 1.1 mL dimethyl sulfoxide (DMSO) and stirred for 1 h. The mixture was then purged with nitrogen for 3 min to remove oxygen. Then 16 mg azo-*bis* isobutyronitrile (AIBN) was added to the solution and the flask was sealed with parafilm before mixing it for one hour. It should be noted that it was operated to obtain a change in the solution viscosity compared to the bulk solution so that this process became more suitable for thin film coating. The obtained solution was stored in a stained flask, as it is light and heat sensitive. A non-imprinted polymer (NIP) without the AMP particles was prepared similarly, for reference purpose.
- Thin Film MIP Coating: 10 μL of MIP solution was spin-coated on the sensors. The spin coating parameters are crucial for the control of the MIP film thickness and homogeneity, typical values of acceleration 4000 rpm/s and velocity 2000 rpm for 10 s were considered for 500 nm layer thickness. In order to localize the polymeric material onto the sensitive path between IDTs, adhesive tape (Kapton) was used as protection, which was removed immediately after spin-coating.
- Polymerization: The coated sensors were then polymerized at 365 nm UV light for 1 h in a polymerization box with continuous flow of nitrogen.
- Removal of AMP template: The extraction of AMP was done by soaking the sensor in eluting solution of ammonium and methanol (aqueous NH_3_ 100 mM/MeOH, 70:30 *v*/*v*) over one night (except other condition mentioned) in a flask covered with parafilm. The device was then rinsed with deionized water (four times) and methanol, before possible storage at 4 °C.

#### 3.1.3. Buffer and Analog Nucleotides Solutions

In order to assay the specificity for AMP of the AMP-MIP, a set of different nucleoside analogs bearing a phosphate moiety and an adenine or cytosine nucleobase, was previously experimented with high-performance liquid chromatography (HPLC) analysis using cartridges loaded by AMP-based bulk MIP [75,78]. The specificity of the microsensors coated with the MIP thin film was studied by using the AMP target and three compounds selected from this set Adenosine-3′-monophosphate (3AMP), cytidine-5′-monophosphate (CMP) and 2-phosphono methoxypropyl adenine (PMPA). Those four commercial nucleotides are illustrated in Figure 1.

### 3.2. Test Bench

#### 3.2.1. Static and Dynamic Modes of Measurements

The so-obtained chemical films were characterized by mechanical and optical profilometry, as well as with scanning electron microscopy (SEM). Electrical characterization of delay-lines was done after each step of the film state: the bare device, after coating and polymerization, after AMP extraction, then after nucleotide rebinding or re-extraction. These tests are called “static mode” tests as the device was immersed in the solution, and then rinsed and dried, placed in a test cell with surrounding ambient air, after which the insertion losses (S21 parameters) are measured in gain and phase with a network analyzer (Anritsu MS4623B, Kanagawa, Japan) as described for example in [79]. On the resulting curves, frequency shifts can be easily measured with the shift on the phase curve, and with the curve of the bare device as a reference. A shift of the frequency can be observed also on the gain curve, as well as variation of the minimum value of insertion losses induced by attenuation of the elastic wave amplitude when the absorption in the sensitive film is modified.

Real-time detection tests of nucleotides have been performed in aqueous media. The acoustic delay-line was associated to a microfluidic chip of polydimethylsiloxane (PDMS) and inserted in an oscillation loop with high short-term stability due to the high quality factor of the LW device, as previously described [80,81]. This typical approach based on real-time monitoring the oscillator frequency allows follow-up of the wave phase velocity with high resolution. By this way, real-time responses of several MIP-coated sensors to various concentrations of AMP and to the analog nucleotides have been investigated.

#### 3.2.2. Microfluidic Set-up

**Figure 2:**
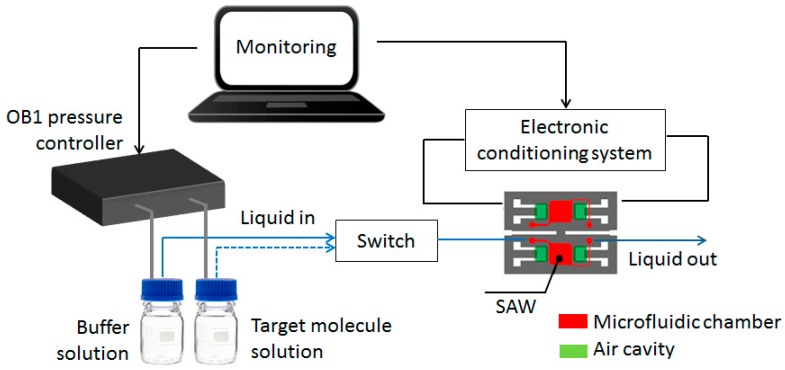
Description of the experimental monitoring setup associated to the LW sensor: a pressure controller (OB1 Elveflow) linked to an actuated valve injects the selected solution into the microfluidic chip, while a frequency counter measures the oscillation frequency as the output signal.

To generate a stable and pulseless flow while remaining extremely reactive we replaced the previously used multi-syringe set by the new flow controller OB1 MkII of Elveflow^®^ (ELVESYS^®^ group, Paris, France) that can control four channels independently for a wide variety of advanced microfluidic applications. The instrument is connected to a dry, dust and oil free pressure source. The pressure and flow rate are computer-controlled using the Elveflow Smart Interface. This software enables to create and run sequences with a specific pressure or flow rate profile. Each pressure outlet can be set independently in a range from 0 up to 200 mbar. The liquids are then pressurized inside flasks with OB1 MkII pressure controller. Pressurized liquids are smoothly and precisely injected onto the PDMS microfluidic chip according to the set pressure or flow rate profile. In our experiments we used two liquids (buffer solution and nucleotides solution), a switch placed before the PDMS microfluidic chip selects the desired liquid. The complete set-up is represented in Figure 2.

## 4. Results

### 4.1. AMP-MIP Thin Film Coating

**Figure 3:**
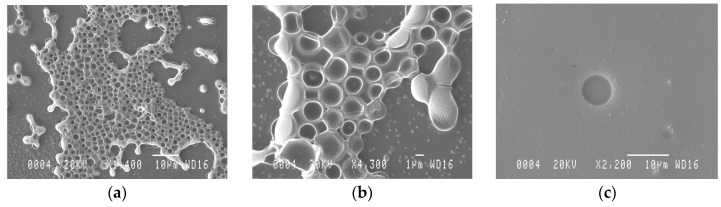
Scanning electron microscopy images of AMP-MIP coatings on the Love Wave sensor surface. (**a**) Molecularly-imprinted polymer; (**b**) molecularly-imprinted polymer at higher magnification; and (**c**) non-imprinted polymer.

Characterization of the so-obtained AMP-MIP coatings revealed typical thicknesses in the range 300–1600 nm, depending on the spin-coater rotation spreading rate, with good surface uniformity and limited to the wave propagation area of the sensor. Microscopy (SEM) in Figure 3a,b revealed the film surface morphology and the pores sizes that range from 100 nm to 5 μm. The absence of pores in the NIP film can be clearly seen on Figure 3c. These images were made on regions typical of the surface of the whole films.

### 4.2. AMP-MIP Thin Film Functional Characterization Based on AMP Detection in Static Mode 

**Figure 4:**
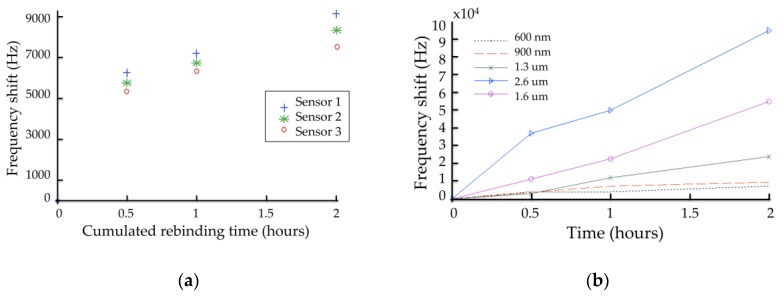
Frequency shift (absolute value) of (**a**) three sensors to 25 ppm of: AMP as target compound, *vs*. the rebinding time (0.5, 1 and 2 h). The sensitive films thickness was 1 µm, the extraction time 3 h; and (**b**) five sensors to 25 ppm of AMP as target compound, vs. the rebinding time (0.5, 1, and 2 h), with the sensitive film thickness as parameter in the range 0.6 µm–2.6 µm.

Detection tests of 25 ppm AMP in static mode, as described previously, were performed with several devices. A concentration of 25 ppm was chosen to obtain easily measurable phase and frequency shifts, although in static mode. Such characterizations in static mode allow more insertion losses due to thick sensitive films up to one or two micrometers. Figure 4a depicts the absolute frequency shifts obtained with three devices coated with a MIP of thickness 1 µm, with 3 h extraction time, as a function of cumulated rebinding time, obtained by successive immersion in AMP solution, followed by the drying step and characterization.

In a similar manner, Figure 4b presents the absolute frequency shifts obtained with five devices coated with AMP-MIP films of thickness in the range from 0.6 to 2.6 µm, with three extraction times, as a function of cumulated rebinding time.

These results demonstrate quite good reproducibility, although the devices were functionalized with the MIP film separately. Longer rebinding times induces higher frequency shifts, demonstrating higher amounts of nucleotides immobilized, as could be expected. With thin MIP layers, below 1 µm, the frequency shift seems to reach a saturation plateau (Figure 4) after 2 h of cumulated rebinding time, leading to shorter duration to reach steady-state, that is to say shorter response time, despite lower sensitivity. Additionally, in a logical way, higher MIP thicknesses lead to higher sensitivity of the sensor, with values as high as 94 kHz at a thickness of 2.6 µm and immersion in 25 ppm AMP solution for 2 h.

### 4.3. Real Time Measurements

However, the final aim is to develop useful devices which require handheld systems with short response times and array facilities. In this approach, a real-time measurement setup was developed, as presented in a previous part, in order to validate the feasibility of such device. Devices used in this part are coated with thin AMP-MIP films of thickness 500 nm, as thicker ones provide higher sensitivity as shown above, but also induce too high insertion losses for proper oscillation. Furthermore, it was shown that thinner films lead to shorter response time.

#### 4.3.1. Detection of AMP

**Figure 5:**
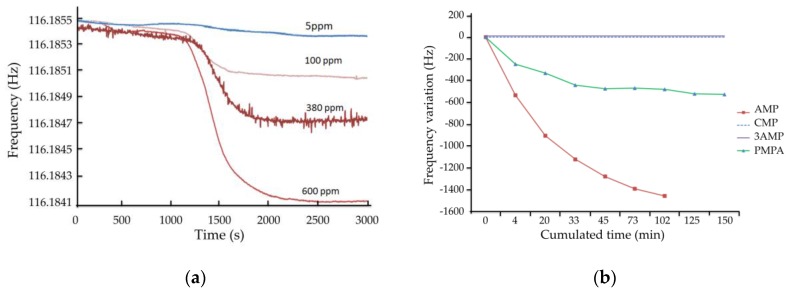
Real-time response of the sensor (**a**) to different concentrations of AMP as target compound; and (**b**) to AMP as target compound and specificity to three similar commercial nucleotides, each one at a concentration of 300 ppm.

The real-time characterization of AMP rebinding is based on the modification of the acoustic wave phase velocity, measured by the oscillator frequency. Figure 5a shows the real-time frequency of the same MIP-coated device for different concentrations of AMP. Between each concentration step, the microsensor was removed from the microfluidic system, immersed in eluting solution and rinsed. It can be observed a dynamic variation of the frequency, starting about one minute after switching from the buffer to the nucleotide solution (transit time in the microfluidic tube) and stabilizing within about 10 min. A steady-state frequency shift of −150 Hz could be observed for 5 ppm AMP concentration, up to −1.4 kHz for 600 ppm. This leads to a sensitivity slightly superior to 2 Hz/ppm (slope of the response above a few ppm). A higher sensitivity at low concentration may be explained by availability of and easy access to the sensitive surface in this case. A detection threshold far better than 1 ppm could then be obtained experimentally, provided that the reference level with buffer solution and the flow rate are well controlled.

Figure 5b represents the response to 300 ppm of AMP. The dynamic protocol was slightly adapted with steps of 4 to 30 min with the sample solution, with intervals of 5 to 20 min with buffer solution, at a flow rate of 5 µL/min. The dynamic frequency shift is represented there as a function of the cumulated time with the sample solution. By doing so, the rebinding effect was increased due to cleaning of non-specific interactions during the process. This resulted in an increased sensitivity, about 5 Hz/ppm for AMP, as well as a longer time before reaching steady-state, as it can be observed when comparing the curves detection of AMP in Figure 5a,b. This experiment was conducted with two sensors functionalized with similar MIP films and led to similar responses (results not shown).

#### 4.3.2. Specificity

Specificity tests were also performed in this dynamic mode. On Figure 5b is superimposed the response to 300 ppm of AMP, as well as the responses to three similar nucleotides 3AMP, CMP, and PMPA, with the same concentration. A binding capacity of the MIP is observed toward PMPA, with a specificity factor of almost three, calculated as the ratio of the steady-state frequency shifts with AMP and PMPA, both of them at the same concentration (300 ppm). No variation was observed with CMP and 3′-AMP. These results are in good agreement with high-performance liquid chromatography (HPLC) analysis previously performed using cartridges loaded by AMP-based bulk MIP [75,78].

**Figure 6:**
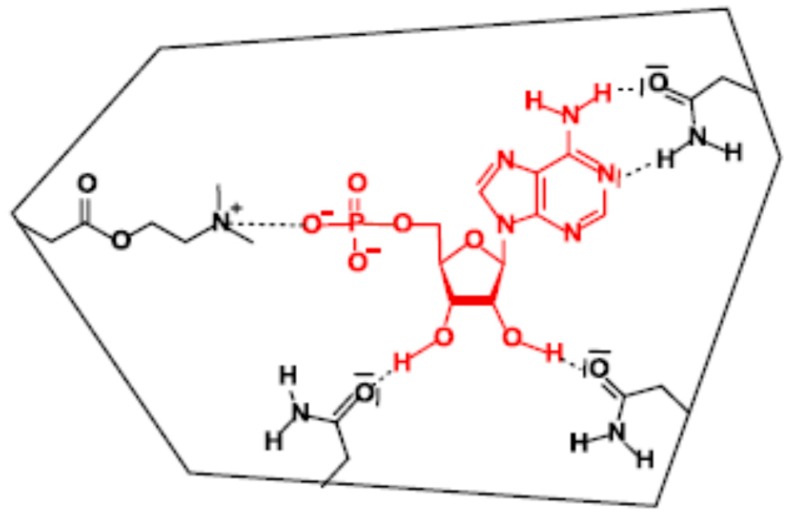
Binding sites in AMP-MIP.

As stated previously, the AMP consists of three components: the phosphate group, the ribose, and the purine base. The selective recognition of AMP in the binding cavity takes place by the participation of all three groups: ionic interaction with DMAEM and phosphate group and hydrogen bonds between AA with ribosyl moiety and adenine base (Figure 6). Moreover, orientation of recognizing sites of these cavities corresponded to those of the binding sites of the template molecule. The MIP selectivity towards other nucleosides is the result of the differences in their structures. The binding site characteristic was performed with frontal analysis, which is an accurate technique for determining adsorption isotherms.

Compared to AMP, the 3′-AMP has a different polarity, lacks one hydroxyl group, and its 3′-phosphate is not in the appropriate orientation to bind to the AMP-MIP cavity. The cytidine 5′-monophosphate (CMP) differs from AMP only by the nucleobase, which cannot reconstitute enough strong and complementary interactions with AA in specific cavity, and is retained on the MIP. Finally, the acyclic AMP analogues, PMPA, can be slightly detected by the AMP-MIP, due to its flexibility, strong binding at the phosphate moiety and at the heterocycle, and despite the lack of ribosyl function.

## 5. Discussions

### 5.1. Results Analysis and Comparison with the State-of-the-Art

Finally, it was shown that LW devices functionalized with MIP films and combined with a microfluidic chip are able to detect, in real-time, low concentrations of nucleoside analogues, compared with static measurements reported in [9], knowing that sensitivity for lower concentrations can be further improved. Moreover, it is known that MIP layers offer a good stability compared to natural recognition reported in [68,82,83]. A good specificity was also obtained, in agreement with that obtained with bulk MIP by cartridge experiments. This agreement is highly interesting, as thin MIP films with thicknesses as low as a few hundreds of nanometers may have had a behavior different from the bulk polymer. Such results pave the way toward MIP coatings based on various nucleosides, such as undergoing experiments with MIP targeting pseudouridine and guanosine, considered as biomarkers for colorectal cancer.

Further experiments should also be undertaken to perform detection and selectivity measurements in real biological samples. However, as mentioned above, various MIP-based sensors have been used to quantify nucleosides in real sample solutions obtained from human fluids (plasma, blood, serum). Generally, it has been shown that the dilution helped mitigate the matrix (proteins, anticoagulant species, and other anions) effect, and that diluted plasma samples were found to be very similar to diluted aqueous samples, in terms of sorption behavior [84,85].

### 5.2. Future Use for Patient-Tailored Chronomodulated Therapy

In addition to that, the way of working of such microsensors is fully adapted to an individual approach of the therapy. Indeed, the chemotherapy of colorectal cancer is based on cytotoxic drugs and therapeutic drug monitoring (TDM) is currently limited by several factors, among them, a considerable intra-individual pharmacokinetic variability [86]. The extremely high sensitivity of such microsensors is based on the reliability of a reference sample, which could, thus, be advantageous while analyzing a small volume of the initial urine sample of the patient. It would result in a quantitative, non-invasive diagnosis tool to monitor the efficacy of chemotherapy in patients with colorectal cancer and to follow up these patients after surgical eradication of their disease, even at a metastatic stage.

Furthermore, this approach could help clinical oncology research related to the circadian timing of cancer therapy, so-called cancer chronotherapeutics [87,88], which provides a possibility to optimize the dose-toxicity relationship through the identification of a temporal window of best tolerability. Indeed, the circadian timing system is composed of molecular clocks, which drive 24-h changes in xenobiotic metabolism and detoxification, cell cycle events, DNA repair, apoptosis, and angiogenesis. As a result, circadian timing can modify two- to 10-fold the tolerability of anticancer medications in experimental models and in cancer patients. This is, to date, an almost unexploited dimension for diagnosis information. Keeping in mind the aim of improved management of patients, the circadian timing approach could then allow the adequate adjustment of treatment delivery to physiological rhythms. Such design of patient-tailored chronomodulated delivery of anticancer medications can further lead to an improved quality of life of patients.

Altogether, a cocktail of MIPs and/or array of microsensing units could lead to an electronic “tongue” approach targeting a disease such as prostate cancer or other type of diseases.

## Abbreviations

The following abbreviations are used in this manuscript:

VOC	volatile organic compound
MIP, NIP	molecularly imprinted polymer, non-imprinted polymer
AMP	adenosine-5′-monophosphate
3AMP	adenosine-3′-monophosphate
CMP	cytidine-5′-monophosphate
PMPA	2-phosphono methoxypropyl adenine
SEM	scanning electron microscopy
DALYs	disability-adjusted life years
IARC	International Agency for Research on Cancer
WHO	world health organization
CT	computed tomography
MRI	magnetic resonance imaging
PET	positron emission tomography
DNA, tRNA	deoxyribonucleic acid, transfer ribonucleic acid
SPE	solid phase extraction
HPLC	high-performance liquid chromatography
UV, IR	ultra-violet, infrared
CE	capillary electrophoresis
LC, GC, MS	liquid chromatography, gas chromatography, mass spectroscopy
FET	field-effect transistor
IDE	interdigitated electrode
FTIR, NDIR	Fourier transform infrared, non-dispersive infrared
SPR	surface plasmon resonance
QCM, SAW	quartz crystal microbalance, surface acoustic wave
LW, SH-SAW	Love wave, shear horizontal surface acoustic waves
PDMS	polydimethylsiloxane
